# Proteome Analysis of Human Sebaceous Follicle Infundibula Extracted from Healthy and Acne-Affected Skin

**DOI:** 10.1371/journal.pone.0107908

**Published:** 2014-09-19

**Authors:** Malene Bek-Thomsen, Hans B. Lomholt, Carsten Scavenius, Jan J. Enghild, Holger Brüggemann

**Affiliations:** 1 Department of Biomedicine, Aarhus University, Aarhus, Denmark; 2 Department of Molecular Biology and Genetics, Aarhus University, Aarhus, Denmark; University of Ulster, United Kingdom

## Abstract

Acne vulgaris is a very common disease of the pilosebaceous unit of the human skin. The pathological processes of acne are not fully understood. To gain further insight sebaceous follicular casts were extracted from 18 healthy and 20 acne-affected individuals by cyanoacrylate-gel biopsies and further processed for mass spectrometry analysis, aiming at a proteomic analysis of the sebaceous follicular casts. Human as well as bacterial proteins were identified. Human proteins enriched in acne and normal samples were detected, respectively. Normal follicular casts are enriched in proteins such as prohibitins and peroxiredoxins which are involved in the protection from various stresses, including reactive oxygen species. By contrast, follicular casts extracted from acne-affected skin contained proteins involved in inflammation, wound healing and tissue remodeling. Among the most distinguishing proteins were myeloperoxidase, lactotransferrin, neutrophil elastase inhibitor and surprisingly, vimentin. The most significant biological process among all acne-enriched proteins was ‘response to a bacterium’. Identified bacterial proteins were exclusively from *Propionibacterium acnes*. The most abundant *P. acnes* proteins were surface-exposed dermatan sulphate adhesins, CAMP factors, and a so far uncharacterized lipase in follicular casts extracted from normal as well as acne-affected skin. This is a first proteomic study that identified human proteins together with proteins of the skin microbiota in sebaceous follicular casts.

## Introduction

In recent years the human microbiota and its modulation of the host inflammatory response have become the subject of extensive research. Whereas the contribution of the gut microbiota in stabilizing or deteriorating the intestinal mucosa and in affecting inflammatory responses in the gut as well as at distant sites starts to unfold, the role of the skin microbiota is far less understood at present. Members of the microbial community of healthy skin are influenced by environmental and physiological parameters and distinct habitats are associated with their own unique microbiota [1]. The sebaceous follicles of healthy human skin provide a unique environment by secretion of lipid-rich sebum, which preferentially admit the growth of bacteria of the genus *Propionibacterium,* in particular *Propionibacterium acnes* (*P. acnes*) [1], [2]. This close association suggests that *P. acnes* acts as a host guardian of the skin [3], [4]. On the other hand, the organism is also associated with acne vulgaris, a frequent chronic inflammatory disease of the sebaceous human follicles that is influenced by genetic host factors, sebum levels and androgens [5]. The role of *P. acnes* in the pathogenesis of acne is continuously discussed, i.e. it is still debated if the microorganism is an innocent bystander or a crucial factor for induction and maintenance of the inflammatory process or even in the development of non-inflammatory elements [6], [7]. A number of *in vitro* studies have shown that *P. acnes* is a potent inducer of inflammation in monocytes, keratinocytes and sebocytes partly through activation of TLR2 and the subsequent release of cytokines and partly through intracellular uptake and activation of the inflammasome [8]–[14]. Also, stimulation through a number of other receptors and pathways such as TLR-4, TLR-9, NOD1, CD36, CRH-R, PAR-2, IGF and ROS are suggested to play a role [15]–[17]. Phylogenetically distinct groups of *P. acnes* have been described, including types IA, IB, IC, II and III; these are further subdivided into Clonal Complexes (CC), based on Multi Locus Sequence Typing (MLST) [18]–[23]. Interestingly, some of these phylotypes were preferentially associated with acne-affected skin, while others were associated with healthy skin or deep tissue infections [20]–[24]. Sequencing of *P. acnes* genomes and comparative genome analyses have identified putative pathogenic traits and genetic differences between phylotypes, which may explain the different roles of *P. acnes* in health and disease [20]–[29]. However, the exact biological significance of these factors is still not known.

A previous study reported gene expression differences between skin biopsies from acne patients and healthy individuals [30]. Many genes involved in pathways associated with inflammation and extracellular matrix remodeling were up-regulated in acne lesions, including genes encoding for matrix metalloproteinases, cytokines (in particular IL-8), and antimicrobial peptides. However, no information about the protein content of normal and acne-affected sebaceous follicle infundibula (SFIs) is available. Thus, the present proteomic study was undertaken to increase our understanding of the processes in the pilosebaceous unit in normal and acne-affected skin *in vivo*. We could identify both human and bacterial proteins produced in SFIs extracted from healthy and diseased skin.

## Results

### Sample selection and methodology pipeline

Human SFIs were extracted by cyanoacrylate-gel (CAG) biopsies from normal skin (nose) of 18 healthy individuals and from acne-affected skin of 20 patients (Table S1). Four different sets of SFI samples were proteome-analyzed (Table 1), including two independent sets of clinical samples from acne-affected facial and back areas of 10 patients (patient groups A1 and A2, sample sets are designated acne-A1 and acne-A2, respectively). From the first group of patients (A1), samples were also collected from the nose (designated nose-A1), that did not show visible signs of acne. Another set of 18 SFI samples were taken from the noses of healthy individuals (designated nose-H1). The number of extracted SFIs differed per person (Table S1); moreover each SFI differed in size and weight. Protein quantification was hampered by sample size limitation and the insoluble sebum-rich material. Thus, for this proof-of-principle study, we decided to pool all extracted SFIs of each individual before processing (see also ‘limitations’ section).

**Table 1:** Information on the four samples sets used for the sebaceous follicular cast proteome project.

Sample name	Patient group(and number)	Age mean(min–max)	Sample site	Leeds score Mean(min–max)	Gender
Acne-A1*	A1 (10)	18.3 years (14–22)	face 7, back 3	4.6 (2–7)	8 male, 2 female
Acne-A2*	A2 (10)	19.7 years (15–24)	face 10, back 1	4.3 (1–7)	5 male, 5 female
Nose-A1*	A1 (10)	18.3 years (14–22)	nose	4.6 (2–7)	8 male, 2 female
Nose-H1*	H1 (18)	30.7 years (25–57)	nose	-	7 male, 11 female

*see also Table S1 for detailed information.

**Figure 1:**
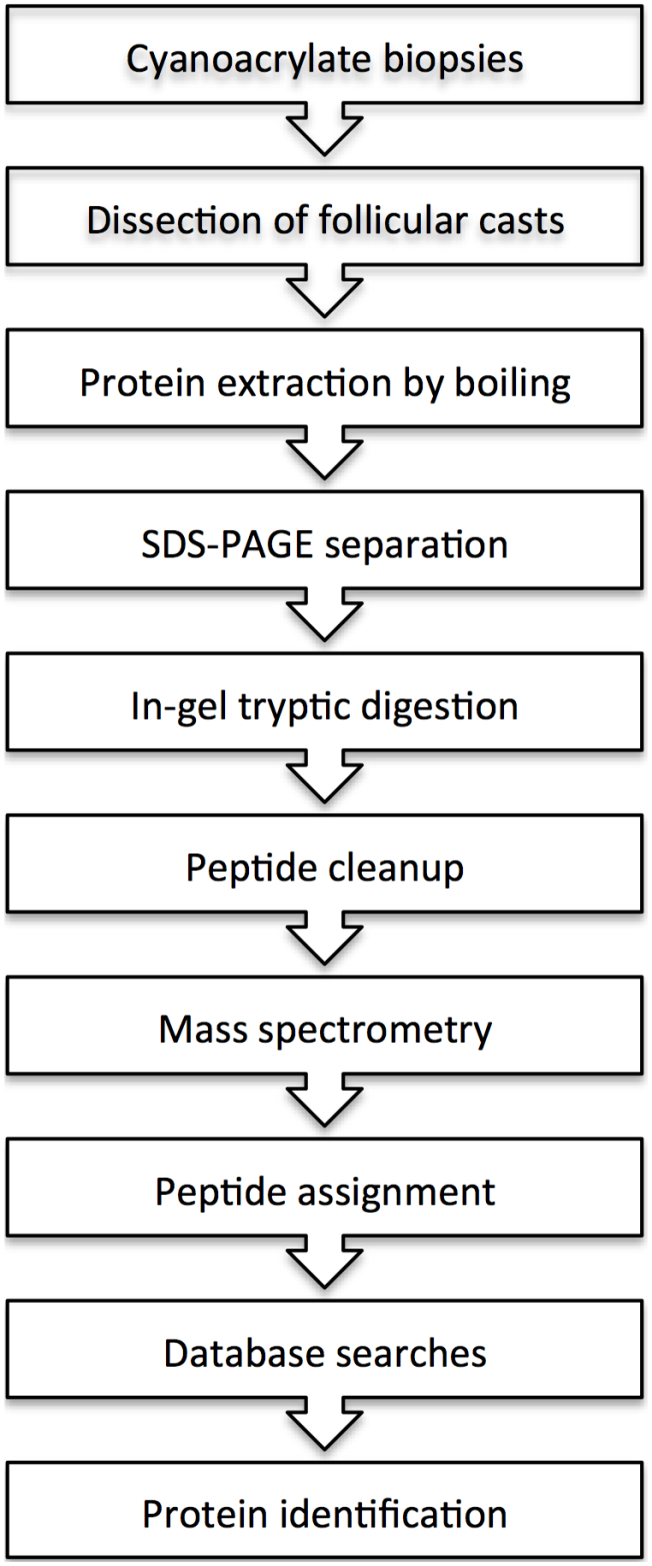
Experimental pipeline of processing extracted human follicular cast material for mass spectrometry analysis.

Three methods of processing the follicular casts were tested, a method that includes the homogenization of SFI material by a FastPrep machine, by a micro-homogenizer and a boiling extraction method, respectively. The boiling extraction method gave most robust results in terms of reproducibility, since loss of material during sample procession was limited. A scheme summarizing the sample processing steps is shown in Figure 1. Protein identification was done by searching the generated MS data against the UniProt database. The boiling method had the possible disadvantage that MS-based protein identification focuses primarily on secreted and soluble proteins in the SFIs; cytoplasmic protein might be underrepresented. Furthermore, the CAG extraction method has drawbacks that are mentioned in the ‘limitations’ section below.

### Human proteome of follicular casts extracted from acne-affected and normal skin

In total, 207 and 238 human proteins were identified in 10 SFI samples of the nose (nose-A1) and 10 samples of acne-affected skin areas (acne-A1), respectively (Tables S2A and S2B). As expected, keratins were the most abundant proteins in the SFI material; a set of keratins including KRT1, KRT6A, KRT6B, KRT5, KRT14 and KRT17 were detected in all samples. KRT1 was used to semi-quantify the protein content per samples: the number of MS-identified KRT1 peptides per sample is given in Table S1; between 22 and 62 KRT1 peptides in samples derived from acne-affected areas (acne-A1) and 28 to 57 peptides in those from nasal areas (nose-A1) could be identified. There is no correlation between the number of processed SFIs and MS-identified KRT1 peptides per sample. This indicates that there is no protein loading limitation also in samples with few SFIs. Overall, there are slightly less KRT1 peptides identified in acne samples (429 peptides) compared to nasal SFI samples (458 peptides), indicative of a slightly lower protein load in acne-A1 compared to nose-A1 samples.

**Figure 2:**
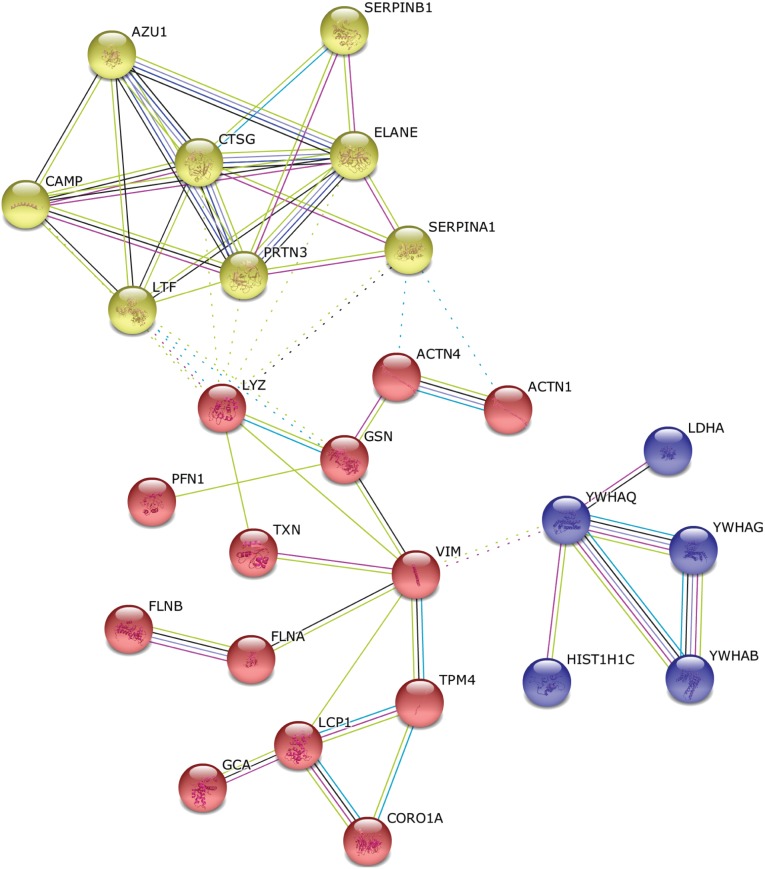
Interaction analysis of human proteins enriched in follicular casts extracted from acne-affected skin sites. The human proteins that are enriched in acne-affected samples (acne-A1) compared to unaffected nasal follicular casts (nose-A1) were analyzed for known protein-protein interactions with the STRING program. The analysis highlighted a cluster of proteins involved in inflammation and host response to a bacterial impact (in yellow) and a cluster of actin-related functions involved in tissue remodeling and repair (in red). A small cluster (in blue) comprises mainly proteins of the 14-3-3 protein family which mediates signal transduction. The complete list of enriched proteins can be found in Tables S2C; a selection is listed in Table 2.

We searched for major differences between the proteome of pooled SFIs from acne-affected versus nasal skin areas. Thus, we focused on the proteins that were unique or strongly overrepresented in acne-A1 or nose-A1 samples: 39 and 22 proteins were strongly enriched in acne-A1 and nose-A1 samples, respectively (Table S2C, Table 2). As a threshold, the respective protein had to be detectable in at least 40% of the samples and had to be detectable in less than 10% in samples of the other set. The 39 acne-enriched SFI proteins, including 12 proteins that were not detected in nose-A1 samples, were analyzed for enriched biological processes based on gene ontology, protein-protein interaction data, and other existing knowledge from functional studies, using the STRING program [31]. A few clusters of functionally related proteins are detectable (Figure 2). One cluster consists of filament and/or actin-interacting proteins, such as the type III intermediate filament protein vimentin (VIM), the actin interacting-proteins filamin A (FLNA), filamin B (FLNB), profiling 1 (PFN1) and the alpha actinins ACTN1 and ACTN4. These factors are associated with processes such as tissue remodeling and wound healing. Another cluster of related proteins contains factors directly or indirectly associated with antimicrobial activity and inflammation including lysozyme (LYZ), lactotransferrin (LTF), cathepsin G (CTSG), cathelicidin antimicrobial peptide (CAMP), azurocidin (AZU1), neutrophil elastase (ELANE) and serpin peptidase inhibitors (SERPINA1, SERPINB1). Accordingly, gene ontology analysis highlighted related enriched biological processes in the acne-A1 data set (table S3): most significant were biological processes related to the response or defense to a bacterium; the process “response to bacterium” (GO:0009617) was highly enriched (p- value 1.52E-9), which included the above mentioned proteins and, in addition, annexin A3 (ANXA3), glutathione S-transferase P (GSTP1) and lipocalin 2 (LCN2). Actin filament-based process (GO:0030029) was another highly enriched (p-value 1.45E-7) biological process, including the proteins VIM, FLNA, FLNB, PFN1, ACTN1, ACTN4, Plastin-2 (LCP1) and gelsolin (GSN).

**Table 2:** Selection of enriched proteins in follicular casts extracted from acne-affected and healthy skin.

Gene name	Function	# acne samples^1^	# healthy samples^2^
MPO	Myeloperoxidase	15	-
SERPINB1	Neutrophil elastase inhibitor	15	-
VIM	Vimentin	13	-
LTF	Lactotransferrin	13	-
ANXA3	Annexin A3	13	2
LCN2	Lipocalin 2	12	2
PFN1	Profilin 1	11	1
CTSG	Cathepsin G	10	-
SERPINA1	Serpin peptidase inhibitor, clade A, member 1	10	-
HBB	Hemoglobin, beta	10	-
LYZ	Lysozyme	9	-
LCP1	Lymphocyte cytosolic protein 1 (L-plastin)	8	-
HBA2	Hemoglobin, alpha 2	8	-
TKT	Transketolase	8	-
APOA1	Apolipoprotein A-I	8	-
FGB	Fibrinogen beta chain	8	-
FGG	Fibrinogen gamma chain	8	-
CORO1A	Coronin, actin binding protein, 1A	8	-
FGA	Fibrinogen alpha chain	8	1
C3	Complement component 3	8	1
ANXA5	Annexin A5	8	2
CTSA	Cathepsin A	2	12
PHB2	Prohibitin 2	-	10
ANXA4	Annexin A4	2	8
HSPE1	Heat shock 10 kDa protein 1 (chaperonin 10)	2	7
PHB	Prohibitin	1	6
PRDX2	Peroxiredoxin 2	2	6

1 sample sets acne-A1 (n = 10) and acne-A2 (n = 10); total number of SFIs = 143.

2 sample set nose-H1 (n = 18); total number of SFIs = 203.

Next, we wanted to confirm and extend the results by analyzing a second set of samples; this time SFIs extracted from a second group of acne patients (acne-A2) were compared to SFIs extracted from the nose of healthy individuals (nose-H1). 302 and 322 proteins were identified in the acne-A2 and nose-H1 samples, respectively, and 30 and 20 proteins were strongly enriched in the acne-A2 and nose-H1 samples, respectively (tables S2D, S2E and S2F). 13 of the 30 acne-A2-enriched proteins were also found to be enriched in the previously analyzed acne-A1 samples. A String analysis highlights a cluster of interacting proteins related to defense and inflammation, similar to the previously identified cluster (Figure 2), including LYZ, LTF, CTSG, SERPINA1, SERPINB1, myeloperoxidase (MPO), apolipoprotein A-I (APOA1), complement component 3 (C3), and fibrinogen (FGA, FGB, FGG). In analogy with the previous analysis, also the second most enriched class of proteins included actin-interacting proteins, such as VIM, LCP1, PFN1, moesin (MSN) and coronin 1A (CORO1A).

Thus, these two independent proteome analyses of SFIs extracted from acne-affected and nasal skin areas gave similar results. The up-regulation of inflammation-associated genes in acne affected skin sites has been shown by a previous gene expression analysis [30]. To identify regulators that govern the changes in the SFI proteins of acne-affected skin, we carried out an Ingenuity Pathways Analysis (IPA). Interestingly, most enriched proteins can be connected via their regulators: figure S1 shows that the production of most proteins enriched in SFIs extracted from acne-affected skin is governed by the transcriptional regulators NF-kB, JNK, PI3 K and/or AKT.

We next analyzed the proteins enriched in the SFIs of the nose (20 proteins in nose-H1 and 22 proteins in nose-A1) compared to the acne samples (acne-A1 and acne-A2) (tables S2C and S2F). Six proteins were commonly enriched in both nasal sample sets: cathepsin A and B (CTSA, CTSB), aspartylglucosaminidase (AGA), peroxiredoxin 1 and 2 (PRDX1, PRDX2) and annexin A4 (ANXA4). CTSA, CTSB and AGA have important roles in lysosome functionality, and the two peroxiredoxins are antioxidant enzymes which reduce hydrogen peroxide and alkyl hydroperoxides. These proteins are included in a cluster of 14 interacting proteins, identified by a STRING analysis (figure S2). No biological process was significantly enriched; however, the KEGG pathway “Lysosome” was enriched (p- value 6.93E-3; data not shown).

Next, we compared the proteomes of nasal SFIs taken from nasal areas of healthy individuals (nose-H1) and acne patients (nose-A1). Even though the nose surfaces of acne sufferers were not visibly affected by disease, there might be changes due to the acne-affected, inflamed facial neighboring environment. Indeed, the comparison highlighted a few differences: the inflammation-induced protein myeloperoxidase (MPO) was detected in 70% of nose-A1 samples but not in nose-H1 samples (tables S2A and S2D, respectively). The other two most prominent factors enriched in nose follicles of acne sufferers are filaggrin 2 (FLG2) and S100 calcium binding protein A7A (S100A7A), both of which belong to the S100 protein family of calcium-regulated proteins that regulate fundamental biological processes. In the proteome of SFIs from noses of healthy individuals (nose-H1) two related proteins are abundant that were absent in the other samples: prohibitin (PHB) and prohibitin 2 (PHB2) (Table 2). Prohibitins play a role both in cell cycle regulation and in maintaining mitochondrial integrity [32].

### Secreted and surface-exposed proteins of *P. acnes* in human follicular casts

Generated MS data were searched against the UniProt database that contains among others also genome-sequenced bacterial species. To avoid the detection of false positives, identification requirements were a MASCOT score above 50 and the detection of at least 2 peptides per protein. The analysis revealed only proteins of *Propionibacterium acnes* in the investigated four data sets (Table S4). In total, 19 *P. acnes* proteins were detected in the 48 examined SFI samples (Table 3). A set of five proteins was identified in >15% of all samples analyzed, thus representing the more abundant proteins of *P. acnes*. The most abundant proteins, i.e. DsA1 and DsA2 and CAMP factor 1 (see below), were detected in the majority of nasal skin samples but were more variably present in disease-associated samples. In general, more *P. acnes* proteins were identified in health-associated samples than in SFI samples obtained from acne-affected skin. We could not identify any *P. acnes* protein that was exclusively present in SFIs from acne-affected skin sites (acne-A1, acne-A2) compared to healthy SFIs (nose-H1) and neither did we observe any *P. acnes* protein solely associated with healthy individuals.

**Table 3:** Proteins of *P. acnes* identified in human follicular casts by mass spectrometry.

Accesion no.(KPA171202)	Product	Healthy*	Patients*		Total*
		Nose-H1 (n = 18)	Nose-A1 (n = 10)	Acne-A1+A2 (n = 20)	n = 48
PPA2127	DsA1; Adhesin/S-layer protein	100 (18)	80 (8)	60 (12)	79 (38)
PPA2210	DsA2; Adhesin/S-layer protein	72 (13)	30 (3)	20 (4)	41 (20)
PPA1340	CAMP factor 1	72 (13)	20 (2)	10 (2)	35 (17)
PPA1796	GehB; triacylglycerol lipase	50 (9)	30 (3)	10 (2)	29 (14)
PPA0644	Putative endoglycoceramidase	33 (6)	10 (1)	7 (1)	16 (8)
PPA2106	Putative endoglycoceramidase	22 (4)	10 (1)	0 (0)	10 (5)
PPA2141	Hypothetical protein	5 (1)	20 (2)	7 (1)	8 (4)
PPA0816	Glyceraldehyde 3-phosphate dehydrogenase	11 (2)	0 (0)	10 (2)	8 (4)
PPA2105	GehA; triacylglycerol lipase	16 (3)	0 (0)	0 (0)	6 (3)
PPA0687	CAMP factor 2	11 (2)	0 (0)	0 (0)	4 (2)
PPA0545	Enolase	5 (1)	0 (0)	5 (1)	4 (2)
PPA1498	Hypothetical protein	11 (2)	0 (0)	0 (0)	4 (2)
PPA2220	Conserved protein	11 (2)	0 (0)	0 (0)	4 (2)
PPA2134	Starvation-inducible DNA-binding protein orfine tangled pili major subunit	5 (1)	0 (0)	5 (1)	4 (2)
PPA1939	Hypothetical protein	5 (1)	0 (0)	0 (0)	2 (1)
PPA1018	Conserved protein	0 (0)	10 (1)	0 (0)	2 (1)
PPA2142	Putative lysophospholipase	5 (1)	0 (0)	0 (0)	2 (1)
PPA1740	Malate dehydrogenase	5 (1)	0 (0)	0 (0)	2 (1)
PPA1873	Elongation factor Tu	5 (1)	0 (0)	0 (0)	2 (1)

*Only proteins identified by more than one peptide are listed. Numbers of positive samples are in percent. Brackets indicate the actual number of samples found to be positive. See table S4 for a detailed list. As reference genome *P. acnes* KPA171202 was used.

Dermatan-sulphate adhesins: The most abundant *P. acnes* factors found in the present study were two proteins designated dermatan-sulphate adhesins 1 and 2, DsA1 (PPA2127) and DsA2 (PPA2210), respectively [21], [33]. Figure S3 shows all MS-identified peptides of DsA1 and DsA2; only the central regions of the two paralogous proteins were MS-identified but not the N- or C-termini. DsA1 was found in all healthy samples (nose-H1) and constituted 80% of the nasal skin samples (nose-A1) and 60% of the acne affected skin samples obtained from patients (Table 3). DsA2 was found in 72% of all healthy (nose-H1) and in 20% of acne-affected skin samples.

CAMP factors: Interestingly, CAMP (Christie-Atkins-Munch-Peterson) factor 1 and 2 were among the *P. acnes* proteins most frequently detected in our samples. CAMP1 was identified in 72% of all healthy individuals (nose-H1), but only identified in 10% of acne-affected skin samples from patients. CAMP2 was found in 14% of healthy samples and not in samples collected from patients (Table 3).

Lipolytic and other hydrolytic enzymes: In agreement with the known lipolytic activity of *P. acnes*, we identified lipolytic enzymes in both health- and disease-associated samples. Surprisingly, the major lipase found *in vivo* was not the characterized triacylglycerol lipase GehA (glycerol-ester hydrolase A, PPA2105). Instead, a so far not studied lipase of *P. acnes* (PPA1796; proposed gene name GehB) was found in 50% of all healthy individuals (nose-H1) and 10% of acne-affected skin samples (acne-A1 and acne-A2).

Host component-degrading enzymes of *P. acnes* have previously been described and suggested playing important roles in the acquisition of nutrients. Two putative endoglycoceramidases (PPA0644 and PPA2106) were detected in the present study. PPA0644 was detected in 33% and 7% of samples extracted from healthy and diseased individuals, respectively. The protein is supposed to catalyse the hydrolysis of the glycosidic linkage between oligosaccharides and ceramides of glycosphingolipids [34].

## Discussion

This study reported a proteomic analysis of human follicular casts extracted from nasal and acne-affected skin. On the host side, human proteins specifically found or enriched in SFIs extracted from acne-affected patients are mainly linked to pathways involved in host responses to bacteria and in tissue repair and regeneration. The production of most of the enriched human proteins in acne samples is governed by the transcriptional regulators NF-kB, JNK, PI3 K and/or AKT (Figure S1).

We could detect microbial proteins in SFIs, but only from one species, *P. acnes*. Our data indicates that SFIs from healthy nasal skin contain *P. acnes* proteins more abundantly than those extracted from acne-affected skin. This trend may be due to the decrease of the *P. acnes* population when the inflammatory response sets in, as previously suggested [35]. Also *P. acnes* may down-regulate its metabolism and protein synthesis, when facing the host inflammatory response. Moreover, most recruited patients were previously treated with antibiotics, which could have changed the microbiota in the pilosebaceous unit. However, clear differential quantification of bacterial proteins found in SFIs from acne and healthy samples, respectively, is hampered by difficulties to quantity the protein content in SFIs and the fact that only a subfraction of extracted follicular casts are colonized [36], [37] (see ‘limitations’ section).

In SFIs extracted from healthy and diseased skin, DsA1 and DsA2 were the most abundant *P. acnes* proteins. The homologs DsA1 and DsA2 (65% protein identity) have been described as host cell-surface attachment proteins with immunoreactive properties [33]. DsA1 and DsA2 are produced by type IA *P. acnes* strains; additional data indicates that neither strains of type IB, type II nor type III can produce DsA1 or DsA2 [21], [33]. This has been explained with a phase variation-like regulation of gene expression, due to a variable C-rich region located just upstream from a putative signal peptide cleavage site. A variation of the C-stretch can result in frameshift mutations. Highly variable is also a hydrophilic proline-threonine (PT)-rich repeat region near the C-terminus that is predicted to be immunogenic and thus, may account for antigenic variation [21], [33]. Since DsA1 and DsA2 were abundantly found in the follicular cast proteome, we conclude that the SFIs of healthy as well as diseased individuals are predominately colonized with type IA *P. acnes* strains, in agreement with previous studies [20]–[24]. It is possible that there are important differences among type IA strains that may account for differences in virulence. This needs to be explored in more detail in the future.

We could detect CAMP factors 1 and 2 of *P. acnes* in the follicular casts. CAMP factors exercise hemolytic activity on erythrocytes in association with sphingomyelinase C from *Staphylococcus aureus*, which is exploited in the CAMP test (to identify Group B streptococci). However, the *in vivo* role of CAMP factors remains elusive, despite some indications that they could act as virulence factors [38]–[40]. Their importance is supported by the conservation of all five CAMP paralogs across the different *P. acnes* phylotypes. A recent publication summarized current knowledge about the CAMP factors of *P. acnes* [23]. It was previously reported that CAMP factor expression differs among *P. acnes* strains [38]. Especially, CAMP1 and CAMP2 were highly expressed under *in vitro* conditions and identified as a major surface-attached and secreted factor of *P. acnes*, respectively [38].

The genome of *P. acnes* encodes at least 12 putative lipases, but only two (GehA and GehB) possess a signal peptide for secretion. GehA and GehB are 42% identical on protein level. The previously characterized lipase GehA is thought to be an important virulence factor and the main enzyme responsible for the hydrolysis of sebum triaglycerides, resulting in the production of free fatty acids (FFA) [41]. FFAs are considered as inflammatory agents, to support ductual hypercornification and to increase adhesion between *P. acnes* and cells of the hair follicle, which promote colonization of *P. acnes* [42]. In addition, a *P. acnes* lipase was recognized as a chemotactic factor [43]. Surprisingly, we detected GehA in only 16% of the health-associated skin samples (nose-H1) and in none of the patient samples. This may indicate a less important role played by GehA *in vivo* than hitherto presumed. Our results suggest that GehB has a more important role in *in vivo* settings. The fact that GehB was mainly associated with healthy skin suggests a beneficial effect of this lipase. However, it is possible that different lipases play distinctive roles in regard to health and disease. Thus, further characterization of GehB is needed to establish its exact role in health and disease.

Whereas the presence of inflammation-associated human proteins in SFIs from acne-affected skin was expected, and is in agreement with previous studies [30], the presence of vimentin in most acne but not in healthy samples was surprising. Vimentin is a type III intermediate filament protein usually found in various non-epithelial cells. Vimentin can also be expressed in cells undergoing physiological or pathological changes, such as epithelial to mesenchymal transition (EMT), a critical event in the induction of cell motility [44]. In agreement, it has been shown that vimentin plays a role in repair functions in response to wounding [45]. The cells of origin of vimentin in the SFI material are unknown. Normal keratinocytes are vimentin-negative, at least under normal circumstances. However, they can express vimentin upon exposure of organic pollutants [46]. The possibility exists that keratinocytes are under stress in acne-affected follicles leading to morphological alterations going along with vimentin expression. Alternatively, vimentin might be secreted from invading macrophages [47] or is derived from adjacent dermal telocytes [48]. Interestingly, we have previously shown that *P. acnes* is present intracellularly in vimentin-positive cells in much higher numbers compared to vimentin-negative cells [49]. Induced vimentin expression in cultured keratinocytes leads to a higher cell invasion rate of *P. acnes*. It was concluded that vimentin is important for either the invasion of *P. acnes* or the intracellular persistence of the bacterium [49]. Our previous work also showed that the intracellular presence of *P. acnes* can elicit a delayed but prolonged NF-kB activation, leading to a long-term inflammatory response [49], [50]. Taken together, it is tempting to speculate that the presence of vimentin in SFIs extracted from acne-affected skin is exploited by *P. acnes*, i.e. vimentin-positive cells might be invaded by *P. acnes*. To further elaborate this hypothesis it is crucial to identify the cells of origin of vimentin in follicular casts from acne patients.

Another aspect of our study is the protein content of SFIs from healthy individuals (table S2F). Here, we found the enrichment of proteins such as two peroxiredoxins (PRDX1, PRDX2) and two prohibitins (PHB, PHB2). The former are antioxidant enzymes whose main function is H_2_O_2_ reduction in cells [51]. Bacterial components such as lipopolysaccharide can induce the production of PRDX1 through activation of the p38 MAPK signal pathway [52]. Interestingly, PRDX1 was suggested to counteract NF-KB-mediated responses, since it was found that PRXD1 deficiency increased the nuclear translocation of NF-κB [52]. The major function of the two prohibitins (PHB; B-cell receptor associated protein-32, BAP-32) and prohibitin 2 (PHB2; repressor of estrogen receptor activity REA; BAP-37) is to maintain the functional integrity of the mitochondria and protecting cells from various stresses [32], [53]. Cellular survival is maintained via prohibitin-mediated activation of the Ras-Raf-MEK-Erk pathway. The absence of prohibitins leads to increased generation of reactive oxygen species (ROS) [53]. In summary, proteins enriched in healthy SFIs seem to have various protective functions, including the inactivation of ROS.

### Limitations

This is the first proteomic analysis of follicular casts extracted from human skin. This study has several limitations. CAG-extracted follicular casts from normal and acne-affected skin are heterogeneous: they differ not only in size and weight, but also in the composition and state of health/disease (e.g. level of inflammation). CAG extractions were carried out on a skin area of each individual with comparable size, i.e. 100 mm^2^. However, extractions of the number of SFIs varied between individuals. In addition, comparing the protein content of the SFI material extracted from each individual was hampered by the limited sample material and its lipid-rich composition. Thus, comparative quantitative analyses are limited. Instead, the presence of peptides from the ubiquitous human keratin KRT1 was used as an internal standard. Moreover, only a subfraction of SFIs are colonized with bacteria [36], [37], which might explain the relatively low abundance of *P. acnes* proteins in the samples. To get enough material for sample processing and MS analysis, all SFIs extracted per individual were pooled in this study. Thus, the proteome is an average analysis from a mixture of SFIs in different stages of health and disease. Optimizing the technique to proteome-analyze defined single follicular casts would be a next research aim.

The proteome data was searched against the current UniProt protein database. It cannot be excluded that (microbial) proteins are present in SFIs whose corresponding sequences are not stored in the database. Moreover, we applied a strict detection threshold (minimum 2 peptides per protein, stringent mascot cutoff) in order to avoid the appearance of false positives. Thus, this approach could have missed microbial proteins present in small quantities. Moreover, metabolites of *P. acnes* such as short-chain fatty acids and other non-proteinaceous products could not be detected with the applied method.

The nose was taken as sampling site from healthy controls. The main reason was the easy access to extractable follicular casts. Acne-affected sites of patients included facial and back skin areas, thus represented different skin sites than the controls. We were not able to extract enough SFI material from healthy cheek skin. Sampling sites and methods of skin sample extractions from acne-affected and control skin has previously been discussed. We refer to this debate rather than to repeat it here [54]–[56].

The patients were recruited randomly by a local dermatologist. Most of the patients were previously treated with antibiotics or antimicrobial substances. We cannot exclude that previous treatments had changed the microbiota in sebaceous follicles.

## Materials and Methods

### Patients

A total of 20 patients and 18 healthy controls were recruited in 2013 by contact with a local dermatologist. All patients were diagnosed by the same specialist in dermatology (HBL). Records of disease and medication were obtained, while the use of cosmetic products was not recorded. Acne activity was graded according to standard Leeds score. Samples and available clinical data are summarized in table 1 and table S1.

### Ethics statement

The full study protocol and all procedures were approved by the Ethics Committee of Denmark Region North under the file number N-20120057. All participants provided documented verbal and written consent. For children (15–17 years) both parents were informed about the study, and the child gave verbal and written consent. This consent procedure was approved by the Ethics Committee.

### Sample processing

Sebaceous follicular infundibula (SFIs) were sampled from human skin by cyanoacrylate gel (CAG) biopsy as previously described by Bek-Thomsen et al. [2]. In patients SFIs were sampled from both acne-affected areas of the face and back and from non-affected nasal skin areas, which allowed us to investigate putative differences in sample sites. To ensure sufficient material for mass spectrometry (MS) analysis healthy samples were collected from nasal skin due to the abundance of sebaceous follicles, which ensured consistent and comparable sampling among individuals. After sampling, follicular casts were dissected with a scalpel under a stereomicroscope and pooled into a microcentrifuge tube containing sample buffer (Lammeli buffer, Bio-Rad). Varying numbers of follicular casts were obtained from each sample, spanning from 2–32. Prior to SDS-PAGE samples were boiled for 5 min (Figure 1). The use of SDS-PAGE ensures separation of the lipid-rich substances comprised in the SFIs and the desired proteins. Our choice of sample processing favors the identification of secreted and surface-associated proteins, which potentially interact with the host.

### Mass Spectrometry

The SDS-PAGE separated sample was prepared for MS analysis by in-gel digestion using trypsin as previously described [57]. The tryptic peptides were micro purified using C18 stage tips according to the manufactures instructions (Proxeon, Thermo Scientific). NanoESI-MS/MS analyses were performed on an EASY-nLC II system (ThermoScientific) connected to a TripleTOF 5600+ mass spectrometer (AB Sciex) equipped with a NanoSpray III source (AB Sciex) operated under Analyst TF 1.5.1 control. The trypsin digested samples were suspended in 0.1% formic acid, injected, trapped and desalted isocratically on a trap column (Biosphere C18 column, 5 µm, 2 cm×100 µm I.D; Nano Separations) after which the peptides were eluted onto and separated by an analytical column. The analytical column was a 15 cm pulled emitter 75 µm ID packed in-house with RP ReproSil-Pur C18-AQ 3 µm resin (Dr. Marisch GmbH, Ammerbuch-Entringen, Germany). Peptides were eluted using 250 nl/min and a 50 min gradient from 5% to 35% phase B (0.1% formic acid and 90% acetonitrile).

### Database searches

Peak lists were generated from the collected MS files by the AB Sciex MS Data Converter beta 1.1 (AB Sciex). Peak lists were searched against a non-redundant UniProt database using an in-house Mascot search engine (Matrix Science). Search parameters were: one missed trypsin cleavage site; propionamide as a fixed modification and methionine oxidation as variable modification. Tolerance for MS and MS/MS were set to 10 ppm and 0.2 Da respectively. All search results were imported into MS Data Miner v. 1.2.1. from which a protein lists were extracted [58].

### Protein interaction and pathway analysis

The String tool (http://string-db.org/) was used to visualize relationships between proteins, such as known and predicted protein–protein interactions [31]. This also includes a tool for identifying enriched Gene Ontology (GO) terms in the generated protein data. In addition, data were analyzed through the use of IPA (Ingenuity Systems, www.ingenuity.com) to detect protein-protein connections, biological pathways and upstream regulators.

## Supporting Information

Figure S1 **Pathway analysis of human proteins enriched in follicular casts extracted from acne-affected skin sites.** Ingenuity was used to create this network, based on the proteins enriched in acne-affected samples (Table 2). Associated network functions are “cellular function and maintenance, inflammatory response, cell-to-cell signaling and interaction”. The associated top canonical pathway is “Acute phase response signaling” (p-value 7.5E-07).
(TIF)

Figure S2 **Interaction analysis of human proteins enriched in follicular casts extracted from healthy nasal skin.** The human proteins that are enriched in healthy samples (nose-H1) were analyzed for known protein-protein interactions with the STRING program. The analysis highlighted one cluster; several proteins are involved in lysosome functionality.
(TIF)

Figure S3 **MS-identification of peptides derived from two homologous surface proteins of** ***P. acnes*** **in human follicular cast samples.** The protein sequence of the two paralogous dermatan-sulphate adhesins DsA1 and DsA2 of *P. acnes* are shown as an alignment. Red characters depict the peptides that were identified by MS analysis. The N-termini, containing signal peptides, and the C-termini, containing PT-repetitive regions, were not MS-identified.
(PDF)

Table S1 **Information about all human participants and the extracted sebaceous follicular cast samples of this study.**
(XLSX)

Table S2 **All human proteins identified by MS in follicular casts extracted from healthy nasal and acne-affected skin.**
(XLSX)

Table S3 **Gene ontology term enrichment analysis highlights biological processes associated with proteins found in follicular casts of acne-affected skin sites.**
(XLSX)

Table S4 **Bacterial proteins identified in follicular casts extracted from acne vulgaris patients and healthy individuals.**
(XLSX)
