# Supplementary figures and images for: Proteome Analysis of Human Sebaceous Follicle Infundibula Extracted from Healthy and Acne-Affected Skin
Source: PLoS One. 2014 Sep 19;9(9):e107908. doi: 10.1371/journal.pone.0107908 (PMC4169578; doi:10.1371/journal.pone.0107908)

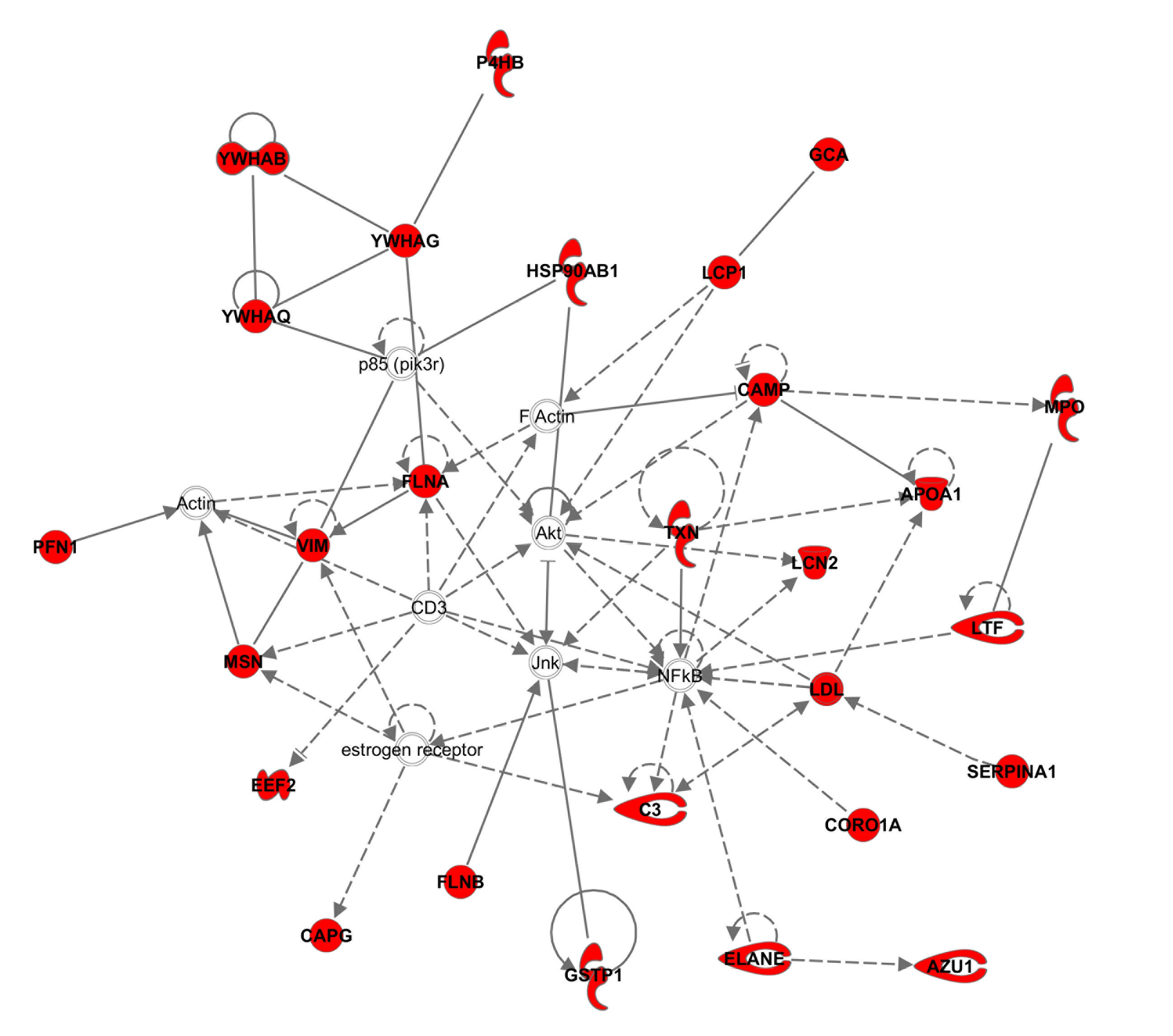

Supplement: Figure S1 — Pathway analysis of human proteins enriched in follicular casts extracted from acne-affected skin sites. Ingenuity was used to create this network, based on the proteins enriched in acne-affected samples (Table 2). Associated network functions are “cellular function and maintenance, inflammatory response, cell-to-cell signaling and interaction”. The associated top canonical pathway is “Acute phase response signaling” (p-value 7.5E-07). (TIF) [file pone.0107908.s001.tif]

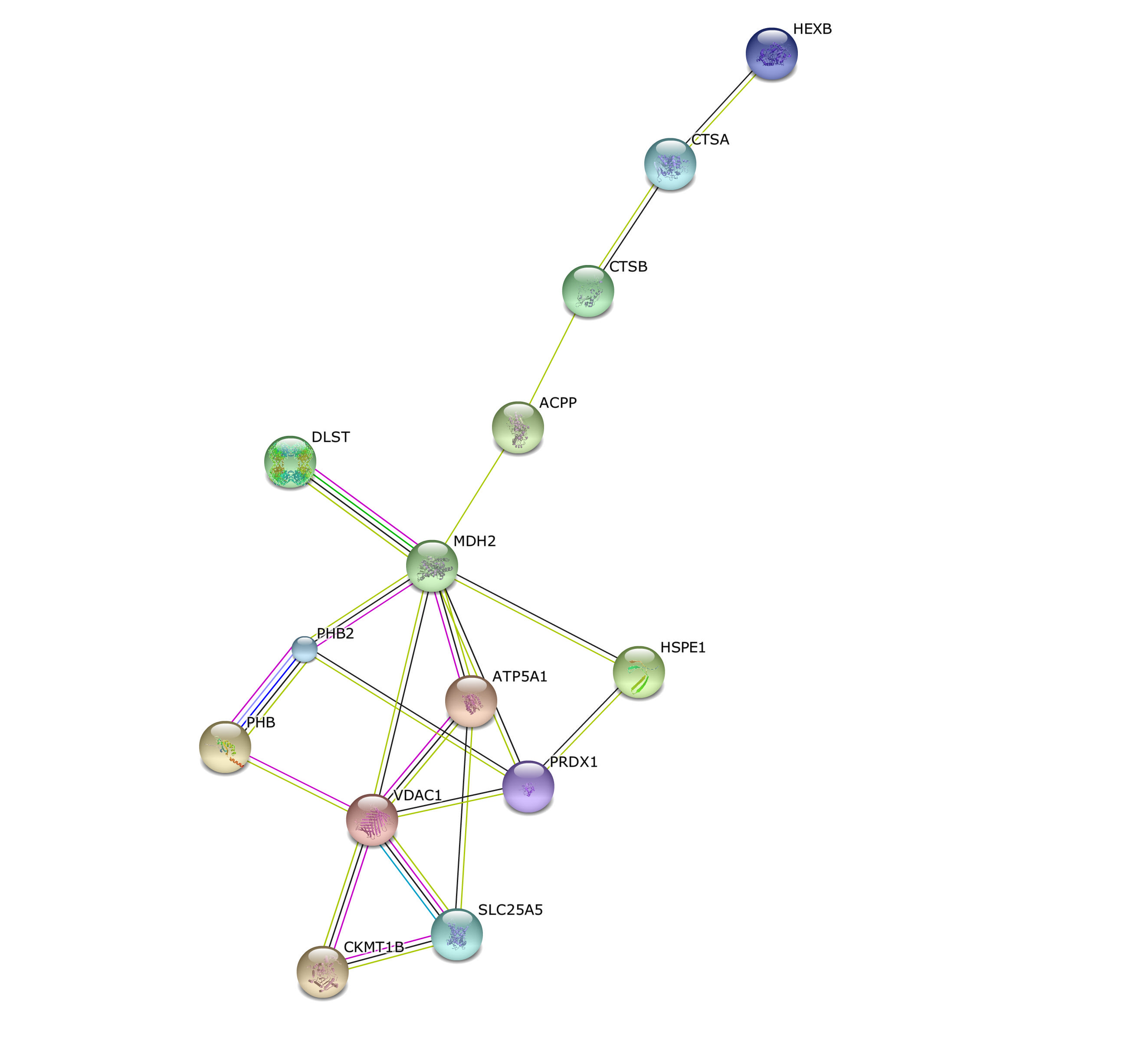

Supplement: Figure S2 — Interaction analysis of human proteins enriched in follicular casts extracted from healthy nasal skin. The human proteins that are enriched in healthy samples (nose-H1) were analyzed for known protein-protein interactions with the STRING program. The analysis highlighted one cluster; several proteins are involved in lysosome functionality. (TIF) [file pone.0107908.s002.tif]
