# Supplementary material for: Proteome Analysis of Human Sebaceous Follicle Infundibula Extracted from Healthy and Acne-Affected Skin
Source: PLoS One. 2014 Sep 19;9(9):e107908. doi: 10.1371/journal.pone.0107908 (PMC4169578; doi:10.1371/journal.pone.0107908)
Supplement: Figure S3 — MS-identification of peptides derived from two homologous surface proteins of P. acnes in human follicular cast samples. The protein sequence of the two paralogous dermatan-sulphate adhesins DsA1 and DsA2 of P. acnes are shown as an alignment. Red characters depict the peptides that were identified by MS analysis. The N-termini, containing signal peptides, and the C-termini, containing PT-repetitive regions, were not MS-identified. (PDF) [file pone.0107908.s003.pdf]

```
DsA1                ------------------MLSLLRRLLRWSDQSQQSPPP----------------------
DsA2                MIIFVRVGNRHMRKRRAVLVVAFQLVNRRLAITKFPTPRRNDASNKNHCGSCDSNCLNRL
                                     :* :  :*:.      : *.*

DsA1                -----PPPQAEASSNRPRSVAQAAIATDGKGIIDKDCRDAVINDAKLRAAIAGALVKAGF
DsA2                TISYHPLPANAASNGNSSITQSAAFSPRATTKISEDCRKAIINDLKLRGAIVGALVKAGL
                         * *   **....  . .**::. ..  *.:***.*:*** ***.**.*******:

DsA1                SSADAVALAPRIAKEMAKEGVLLINHHKLKALIGAQLGLLTDAKIQRAAAAVDLGIKATL
DsA2                SAADAAALAPRIAAEMAAEGTLTINHHRLKVLVASQLGLVADAAVQHAAAAIDLSFKAIL
                    *:***.******* *** **.* ****:**.*:.:****::** :*:****:**.:** *

DsA1                AATIIPNALHSAAFKDAVVANLVAAGVDKKLAKATAVAIAATALNPALGPIAKTEAIKAE
DsA2                GASIIPNALGSAAFKNAVIANLVAAGIDKHLARATAVAIVATALNPALGPIAKFELIKAE
                    .*:****** *****:**:*******:**:**:******.************* * ****

DsA1                IAAQAALLVGRGVHLKKAAIEHIIGRSFDAAVATAIVSSPILNARIVTHLVRAGIDKSLA
DsA2                IAAQAALLIRRGVHLQKAAIEHVIGRAFDAAVATAIISSPILSARIVTHLVRAGIDKSIA
                    ********: *****:******:***:*********:*****.***************:*

DsA1                VQIAPRIIDRLAKEPLLALNTAKLMKNITRQIVDVITADKAIKTAEQLEKELPALDDLVK
DsA2                ISLAPHIVKRLAKEPLLAFNTAKLVKDIARQIVDIRNTQEAIAVYKQLKAELPTLDGLVQ
                    :.:**:*:.*********:*****:*:*:*****: .:::** . :**: ***:**.**:

DsA1                KACS---------------CPKPTPTPTPTPTPKPTPTPTPKPTPTPKPKPTPAPAPT
DsA2                KACTPEPTPTPTPTPTPTPTPAPTPTPAPTPTPAPTPAPTPTPAPTPTPTPTPTPTPTPT
                    ***:                * *****:*****:*.*:***** *****.*.***:*:**

DsA1                SGATSDESTSRSGGHSQGGSGTHYIHHGVAPVLTHSSDLPSTGF--
DsA2                HGATTTTPISRTTDRHNLGS---HHTRIAAPALIHAKALPATGTGA
                     ***:  . **: .: : **   :  : .**.* *:. **:**
```

Figure S3: MS-identification of peptides derived from two homologous surface proteins of *P. acnes* in human follicular samples. The protein sequence of the two paralogous dermatan-sulphate adhesins DsA1 and DsA2 of *P. acnes* are shown as an alignment. Red characters depict the peptides that were identified by MS analysis. The N-termini, containing signal peptides, and the C- termini, containing PT-repetitive regions, were not MS-identified.
